# Coagulation Biomarkers and Clinical Outcomes in Elderly Patients With Nonvalvular Atrial Fibrillation: ANAFIE Subcohort Study

**DOI:** 10.1016/j.jacasi.2023.06.004

**Published:** 2023-08-15

**Authors:** Yukihiro Koretsune, Takeshi Yamashita, Masaharu Akao, Hirotsugu Atarashi, Takanori Ikeda, Ken Okumura, Wataru Shimizu, Shinya Suzuki, Hiroyuki Tsutsui, Kazunori Toyoda, Atsushi Hirayama, Masahiro Yasaka, Takenori Yamaguchi, Satoshi Teramukai, Tetsuya Kimura, Yoshiyuki Morishima, Atsushi Takita, Hiroshi Inoue

**Affiliations:** a National Hospital Organization Osaka National Hospital, Osaka, Japan; b The Cardiovascular Research Institute, Tokyo, Japan; c Department of Cardiology, National Hospital Organization Kyoto Medical Center, Kyoto, Japan; d AOI Hachioji Hospital, Tokyo, Japan; e Department of Cardiovascular Medicine, Toho University Faculty of Medicine, Tokyo, Japan; f Division of Cardiology, Saiseikai Kumamoto Hospital Cardiovascular Center, Kumamoto, Japan; g Division of Cardiology, Nippon Medical School Department of Medicine, Tokyo, Japan; h Department of Cardiovascular Medicine, Kyushu University Graduate School of Medical Science, Fukuoka, Japan; i Department of Cerebrovascular Medicine, National Cerebral and Cardiovascular Center, Suita, Japan; j Osaka Police Hospital, Osaka, Japan; k Department of Cerebrovascular Medicine and Neurology, Cerebrovascular Center, National Hospital Organization Kyushu Medical Center, Fukuoka, Japan; l Department of Biostatistics, Graduate School of Medical Science, Kyoto Prefectural University of Medicine, Kyoto, Japan; m Primary Medical Science Department, Daiichi Sankyo, Tokyo, Japan; n Data Intelligence Department, Daiichi Sankyo Co, Ltd, Tokyo, Japan; o Saiseikai Toyama Hospital, Toyama, Japan

**Keywords:** all-cause death, coagulation biomarker, D-dimer, prothrombin, stroke, systemic embolic events

## Abstract

**Background:**

Little is known about the relationship between coagulation biomarkers and clinical outcomes in patients with atrial fibrillation (AF) treated with anticoagulants, especially direct oral anticoagulants (DOACs) and warfarin.

**Objectives:**

This subcohort study evaluated the association between coagulation biomarkers and clinical outcomes in elderly Japanese patients with nonvalvular AF using the ANAFIE (All Nippon AF In the Elderly) Registry.

**Methods:**

Patients with a definitive diagnosis of nonvalvular AF and aged ≥75 years at enrollment were included. At enrollment, biomarker levels for D-dimer, thrombin–antithrombin complex (TAT), prothrombin fragment 1+2 (F1+2), and soluble fibrin monomer complex (SFMC), along with data on anticoagulant use, were recorded.

**Results:**

Of the 3,194 patients, 95.1% were using oral anticoagulants (OACs) (71.7% DOACs, 23.4% warfarin). D-dimer, TAT, and F1+2 levels, as well as the proportion of patients with a positive SFMC, were lower among those receiving OACs compared with those not receiving OACs. In the DOAC group, higher levels of D-dimer (≥1.0 μg/mL) and TAT (>3 ng/mL) were significantly associated with increased incidences of cardiovascular (CV) events (stroke, myocardial infarction, cardiac intervention, heart failure, and CV death), all-cause death, and CV death. In the warfarin group, higher levels of D-dimer were significantly associated with increased rates of all-cause death, higher levels of TAT with increased major bleeding, and positive SFMC with increased major bleeding and CV events.

**Conclusions:**

Higher levels of coagulation biomarkers were associated with a higher risk of worse clinical outcomes, and the relationships between the coagulation biomarkers and outcomes differed between the DOAC and warfarin groups. (Prospective Observational Study in Late-Stage Elderly Patients with Non-Valvular Atrial Fibrillation All Nippon AF In Elderly Registry-ANAFIE Registry; UMIN000024006)

Atrial fibrillation (AF) is associated with an increased risk of stroke and systemic embolic events (SEE). Although anticoagulant therapy is the recommended primary approach to prevent stroke and SEE in AF,^1^ managing bleeding, the risk of which increases with anticoagulant use, is of prime importance.^2^ Recent large-scale, real-world data from Japan indicate that, among patients with AF on anticoagulant therapy, nearly one-third of the residual risks of ischemic stroke and SEE are attributable to modifiable risk factors, such as hypertension, diabetes, and dyslipidemia.^3^

Optimal anticoagulation is a cornerstone of stroke prevention in patients with AF. However, there is a need to assess modifiable and nonmodifiable risk factors for both stroke and bleeding when initiating antithrombotic therapy.^4^ Currently, the risk of stroke and bleeding in patients with AF is assessed using the CHADS_2_/CHA_2_DS_2_-VASc scores and the HAS-BLED score, respectively.^5^ Performing a more personalized risk assessment for individual patients is currently challenging; however, including data on coagulation biomarkers may allow a more detailed risk assessment of stroke, bleeding, or other outcomes.

Anticoagulant therapy involves the use of direct oral anticoagulants (DOACs) and warfarin, which have different mechanisms of action. Whereas DOACs directly inhibit a single activated coagulation factor (FXa/thrombin) and have no effect on coagulation factor abundance, warfarin inhibits the biosynthesis of vitamin K–dependent coagulation factors (prothrombin, FVII, FIX, and FX), thereby reducing their levels. Furthermore, DOACs and warfarin are suggested to have different effects on coagulation markers in patients with AF.^6^

Phase 3 trials of DOACs, such as the RE-LY (Randomized Evaluation of Long-term Anticoagulation Therapy),^7^ ARISTOTLE (Apixaban for the Prevention of Stroke in Subjects With Atrial Fibrillation),^8^, ^9^, ^10^, ^11^ and ENGAGE AF-TIMI 48 (Effective Anticoagulation with Factor Xa Next Generation in Atrial Fibrillation-Thrombolysis In Myocardial Infarction 48)^12^ trials, indicate that D-dimer, cardiac troponin, and N-terminal pro–B-type natriuretic peptide are predictors of stroke and mortality that significantly enhance predictive ability when added individually to clinical risk scores. However, in most of these trials, patients treated with DOACs or warfarin were not analyzed separately; instead, they were pooled together.

Currently, there is little evidence in which patients with AF receiving DOACs and those with AF receiving warfarin were separately analyzed and compared when examining the association between coagulation biomarkers and clinical outcomes. Although coagulation biomarkers may contribute to more detailed individual efficacy and safety risk assessments,^13^^,^^14^ there is a dearth of data investigating the relationship between coagulation biomarkers and stroke/SEE, major bleeding, mortality, and cardiovascular (CV) events, especially in elderly patients with nonvalvular atrial fibrillation (NVAF) in a real-world setting.

We have previously reported 2-year clinical outcomes for more than 30,000 elderly patients aged ≥75 years with NVAF in the ANAFIE (All Nippon AF In the Elderly) Registry.^15^ The objective of this subcohort study was to investigate the relationship between coagulation biomarkers and clinical outcomes in elderly Japanese patients with NVAF treated with DOACs or warfarin using the ANAFIE Registry dataset.

## Methods

### Study design

The rationale and study design of the ANAFIE Registry (UMIN000024006) have been previously described in full.^15^ Briefly, the ANAFIE Registry was a prospective, multicenter, observational study with a 2-year follow-up. The population (n = 32,275) consisted of elderly Japanese patients (aged ≥75 years) with a definitive diagnosis of NVAF. The ANAFIE Registry was approved by the ethics committees of the participating centers. The study was conducted in compliance with the Declaration of Helsinki, local regulations for registries, and ethical guidelines for clinical studies in Japan. Written informed consent was provided by all participants (or family members for patients with communication disorders, such as aphasia or cognitive impairment) for the primary ANAFIE Registry as well as for this subcohort study.

### Patients

The detailed inclusion and exclusion criteria of the overall ANAFIE Registry have previously been published.^16^ Patients from the ANAFIE Registry who agreed to participate in this study to assess the relationship between coagulation biomarkers and clinical outcomes were included in this subcohort.

### Study measures and endpoints

The following coagulation biomarkers were measured at SRL Medisearch Inc: D-dimer, thrombin–antithrombin complex (TAT), prothrombin fragment 1+2 (F1+2), and soluble fibrin monomer complex (SFMC).

In patients taking DOACs at the time of enrollment, blood samples were collected before taking anticoagulants (during trough) on any day within 90 days from the date of consent acquisition. For those on warfarin at the time of enrollment and those who were not receiving anticoagulants, blood samples were collected at any routine visit within 90 days from the date of consent acquisition. In patients newly started on DOACs or warfarin, blood samples were collected ≥30 days after the start of administration.

The study outcomes during the 2-year follow-up were stroke/SEE, major bleeding, intracranial hemorrhage (ICH), CV events (a composite of stroke, myocardial infarction, cardiac intervention, heart failure, and CV death), CV death, and all-cause death. All events were adjudicated by event evaluation committees. Major bleeding was classified according to the International Society on Thrombosis and Haemostasis definition.

### Statistical analysis

The planned sample size for this subcohort study was 3,000 patients. For this analysis, frequency tables were created for categorical variables and summary statistics were calculated for continuous variables. Where applicable, the frequency or incidence rate/100 person-years of events and their 95% CIs were calculated. The *P* values were calculated using the chi square test for categorical variables, and using analysis of variance for continuous variables. No imputations were made for missing data, which were not included in the analyses.

The biomarkers used in this study were categorized as follows: D-dimer (<1.0 μg/mL [reference]; ≥1.0 μg/mL), TAT (≤3 ng/mL [reference]; >3 ng/mL), F1+2 (<69 pmol/L; 69-<229 pmol/L [reference]; ≥229 pmol/L), and SFMC (positive; negative [reference]). Cutoff values were determined based on the standard values described in the package insert of the reagents used for each of the measurements.

The Cox proportional-hazards model was used to calculate the HR and its 95% CI. Variables considered possibly associated with anticoagulant therapy selection or outcome incidences were used in the statistical model and included the following: sex, age, body mass index, history of major bleeding, type of AF, hypertension, severe liver function disorder, diabetes mellitus, hyperuricemia, heart failure/left ventricular systolic dysfunction, myocardial infarction, cerebrovascular disease, thromboembolic disease, active cancer, dementia, falls within 1 year, catheter ablation, antiarrhythmic agents, antiplatelet agents, proton pump inhibitors, P-glycoprotein inhibitors, dyslipidemia, creatinine clearance, digestive disease, and polypharmacy. Regarding the significance level used, tests were 2-sided, and a *P* value of <0.05 was considered statistically significant. Logistic regression analysis was used to analyze the factors that affect biomarker levels; variables used in the model are listed earlier in this paper (Cox proportional-hazards model). All statistical analyses were conducted using SAS version 9.4 or higher (SAS Institute).

## Results

Of the 32,275 patients enrolled in the ANAFIE Registry, 3,194 patients agreed to participate in this subcohort study. Of these, 20 patients did not have biomarker measurements, resulting in 3,174 total patients. Table 1 describes the characteristics of the patients at the time of enrollment. The majority of the patients (2,290/3,194; 71.7%) in this subcohort were using DOACs, 747 (23.4%) were using warfarin, and 157 (4.9%) were not using any oral anticoagulant (OAC). In this subcohort, 25.6% of patients were ≥85 years of age, with the No-OAC group having a significantly greater proportion of patients aged ≥85 years. In this cohort overall, 46.6% of patients had a creatinine clearance of <50 mL/min, and the proportion of patients with creatinine clearance of <50 mL/min was higher among patients treated with warfarin than with DOACs or with no OACs.

**Table 1:** Patient Characteristics at Enrollment

	Total (N = 3,194)	Warfarin (n = 747)	DOAC (n = 2,290)	No-OAC (n = 157)	*P* Value
Male	1,876 (58.7)	483 (64.7)	1,315 (57.4)	78 (49.7)	<0.001
Age, y	81.4 ± 4.8	81.8 ± 4.8	81.2 ± 4.7	82.4 ± 6.0	<0.001
≥85 y	817 (25.6)	218 (29.2)	544 (23.8)	55 (35.0)	<0.001
Body mass index, kg/m^2^	23.4 ± 3.6	23.5 ± 3.8	23.4 ± 3.5	23.2 ± 3.6	0.671
Systolic blood pressure, mm Hg	127.8 ± 17.0	125.9 ± 17.2	128.2 ± 16.8	130.0 ± 17.1	0.002
Diastolic blood pressure, mm Hg	70.6 ± 11.4	69.8 ± 11.5	71.0 ± 11.4	69.4 ± 11.2	0.027
Creatinine clearance, mL/min	48.0 ± 17.1	43.8 ± 18.3	49.3 ± 16.4	47.0 ± 19.4	<0.001
<50 mL/min	1,490 (46.6)	381 (51.0)	1,045 (45.6)	64 (40.8)	<0.001
CHADS_2_ score	2.9 ± 1.2	3.0 ± 1.2	2.9 ± 1.2	2.8 ± 1.0	0.070
CHA_2_DS_2_-VASc score	4.5 ± 1.4	4.6 ± 1.4	4.5 ± 1.4	4.5 ± 1.3	0.263
HAS-BLED score	1.9 ± 0.9	2.0 ± 0.9	1.9 ± 0.9	2.0 ± 0.9	<0.001
History of major bleeding	143 (4.5)	30 (4.0)	98 (4.3)	15 (9.6)	0.007
AF type					
Paroxysmal	1,293 (40.5)	231 (30.9)	964 (42.1)	98 (62.4)	<0.001
Persistent/long-standing persistent	954 (29.9)	224 (30.0)	691 (30.2)	39 (24.8)	
Permanent	947 (29.6)	292 (39.1)	635 (27.7)	20 (12.7)	
TTR	-	74.6 ± 30.7	-	-	-
PT-INR	-	2.0 ± 0.3	-	-	-
History of nonpharmacological therapy for AF	488 (15.3)	107 (14.3)	355 (15.5)	26 (16.6)	0.666
Catheter ablation	207 (6.5)	30 (4.0)	159 (6.9)	18 (11.5)	<0.001
Comorbidities					
Hypertension	2,463 (77.1)	556 (74.4)	1,779 (77.7)	128 (81.5)	0.074
Diabetes mellitus	942 (29.5)	236 (31.6)	656 (28.6)	50 (31.8)	0.248
Hyperuricemia	756 (23.7)	230 (30.8)	502 (21.9)	24 (15.3)	<0.001
Chronic kidney disease	666 (20.9)	205 (27.4)	432 (18.9)	29 (18.5)	<0.001
Myocardial infarction	180 (5.6)	50 (6.7)	124 (5.4)	6 (3.8)	0.252
Heart failure/left ventricular systolic dysfunction	1,247 (39.0)	326 (43.6)	864 (37.7)	57 (36.3)	0.012
Cerebrovascular disease	787 (24.6)	183 (24.5)	580 (25.3)	24 (15.3)	0.018
Digestive diseases	943 (29.5)	207 (27.7)	676 (29.5)	60 (38.2)	0.032
Active cancer	341 (10.7)	66 (8.8)	259 (11.3)	16 (10.2)	0.161
Dementia	268 (8.4)	54 (7.2)	196 (8.6)	18 (11.5)	0.190
Fall within 1 y	281 (8.8)	75 (10.0)	190 (8.3)	16 (10.2)	0.182

Values are n (%) or mean ± SD.

AF = atrial fibrillation; DOAC = direct oral anticoagulant; OAC = oral anticoagulant; PT-INR = prothrombin time-international normalized ratio; TTR = time in therapeutic range.

The Central Illustration shows the median (Q1, Q3) levels of the biomarkers and the distribution of patients at enrollment according to the use of anticoagulants for the biomarker categories. Significantly higher levels of D-dimer, TAT, and F1+2 were observed among patients who were not receiving anticoagulants compared with patients receiving DOACs (*P* < 0.001 for all biomarkers) or warfarin (*P* < 0.001 for D-dimer and F1+2; *P* = 0.007 for TAT). In addition, patients who were not receiving OACs were significantly more likely to have positive SFMC than patients receiving warfarin (*P* = 0.037). No difference was observed between the DOAC and No-OAC groups in terms of SFMC. Patients receiving DOACs also had higher levels of D-dimer and F1+2 compared with patients taking warfarin; although these reached statistical significance, the median differences were small. Patients receiving warfarin were also significantly less likely to have positive SFMC than patients receiving DOACs (*P* = 0.004). No significant differences between the DOAC and warfarin groups were observed for TAT. Table 2 depicts biomarker levels at enrollment by the type of anticoagulant use. Of note, 32.5% of patients in the No-OAC group had D-dimer ≥1.0 μg/mL, which was approximately 3-fold higher than the proportion of patients using DOACs (10.1%) or warfarin (13.8%). Similarly, a higher proportion of those in the No-OAC group (15.3%) had TAT >3 ng/mL compared with the DOAC (6.4%) and warfarin (6.2%) groups. In the No-OAC group, 75.8% of patients had F1+2 ≥229 pmol/L, compared with only 30.4% in the DOAC group and 11.1% in the warfarin group. The proportions of patients observed to be SFMC positive ranged from 15.0% in the warfarin group to 20.2% in the DOAC group and 22.3% in the No-OAC group. The distribution of each biomarker among patients in the DOAC group that excluded patients receiving an off-label dose was similar to that in the entire DOAC group.

**Central Illustration:**
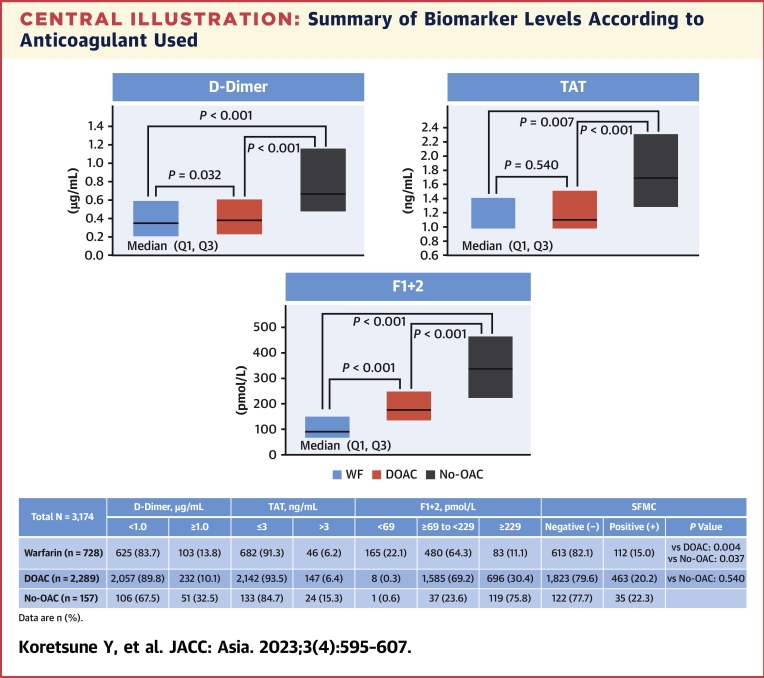
Summary of Biomarker Levels According to Anticoagulant Used Median (Q1, Q3) biomarker levels and the distribution of patients at enrollment according to the usage of anticoagulants for the biomarker categories are shown. Blood was collected at enrollment and biomarker levels were determined. *P* values were calculated using the chi square test for categorical variables, and analysis of variance for continuous variables. Significantly higher levels of D-dimer, TAT, and F1+2 were observed among patients who were not receiving anticoagulants compared with patients receiving DOACs or warfarin. The likelihood of having positive SFMC was higher for patients either not receiving OACs or receiving DOACs vs patients receiving warfarin. DOAC treatment resulted in higher levels of D-dimer and F1+2 versus warfarin treatment. DOAC = direct oral anticoagulant; F1+2 = prothrombin fragment 1+2; OAC = oral anticoagulant; Q = quartile; SFMC = soluble fibrin monomer complex; TAT = thrombin–antithrombin complex; WF = warfarin.

**Table 2:** Biomarkers by Type of Anticoagulant at Enrollment

	No-OAC (n = 157)	Warfarin (n = 728)	DOAC (n = 2,289)	DOAC (Excluding Off-Label Doses) (n = 1,916)
D-dimer (μg/mL)				
<1.0	106 (67.5)	625 (83.7)	2,057 (89.8)	1,721 (89.8)
≥1.0	51 (32.5)	103 (13.8)	232 (10.1)	195 (10.2
TAT (ng/mL)				
≤3	133 (84.7)	682 (91.3)	2,142 (93.5)	1,795 (93.7)
>3	24 (15.3)	46 (6.2)	147 (6.4)	121 (6.3)
F1+2 (pmol/L)				
<69	1 (0.6)	165 (22.1)	8 (0.3)	7 (0.4)
≥69 to <229	37 (23.6)	480 (64.3)	1,585 (69.2)	1,334 (69.6)
≥229	119 (75.8)	83 (11.1)	696 (30.4)	575 (30.0)
SFMC				
Negative	122 (77.7)	613 (82.1)	1,823 (79.6)	1,521 (79.4)
Positive	35 (22.3)	112 (15.0)	463 (20.2)	393 (20.5)

Values are n (%).

F1+2 = prothrombin fragment 1+2; SFMC = soluble fibrin monomer complex; TAT = thrombin–antithrombin complex; other abbreviations as in Table 1.

The relationships between coagulation biomarkers and clinical outcomes in patients using DOACs or warfarin are summarized in Tables 3, 4, 5, and 6. In the DOAC group, higher levels of D-dimer (≥1.0 vs <1.0 μg/mL) and TAT (>3 vs ≤3 ng/mL) were significantly associated with increased incidences of composite CV events (D-dimer: adjusted hazard ratio [aHR]: 1.91; 95% CI: 1.37-2.66; *P* < 0.001; TAT: aHR: 1.60; 95% CI: 1.04-2.46; *P* = 0.033), CV death (D-dimer: aHR: 3.54; 95% CI: 1.75-7.18; *P* < 0.001; TAT: aHR: 4.49; 95% CI: 2.04-9.92; *P* < 0.001), and all-cause death (D-dimer: aHR: 3.20; 95% CI: 2.14-4.78; *P* < 0.001; TAT: aHR: 2.85; 95% CI: 1.74-4.64; *P* < 0.001). In addition, higher levels of F1+2 (≥229 pmol/L vs 69 to <229 pmol/L) were associated with increased incidences of CV events (aHR: 1.31; 95% CI: 1.00-1.71; *P* = 0.0496), and all-cause death (aHR: 1.47; 95% CI: 1.01-2.13; *P* = 0.044) in patients receiving DOACs. In the warfarin group, higher levels of D-dimer were significantly associated with increased incidences of all-cause death (aHR: 2.41; 95% CI: 1.36-4.25; *P* = 0.003), higher levels of TAT with increased incidences of major bleeding (aHR: 14.03; 95% CI: 1.50-131.06; *P* = 0.021), and positive SFMC with increased incidences of major bleeding (aHR: 6.37; 95% CI: 1.31-30.99; *P* = 0.022), and CV events (aHR: 1.69; 95% CI: 1.06-2.72; *P* = 0.029). Furthermore, low levels of F1+2 (<69 pmol/L vs 69 to <229 pmol/L) were associated with a decreased incidence of all-cause death (aHR: 0.21; 95% CI: 0.06-0.70; *P* = 0.011) in patients receiving warfarin.

**Table 3:** Relationships Between D-Dimer and Clinical Outcomes Based on OAC Use

	D-Dimer, μg/mL	Warfarin (n = 728)				DOAC (n = 2,289)			
		n	Incidence Rate, /100 py	aHR (95% CI)	*P* Value	n	Incidence Rate, /100 py	aHR (95% CI)	*P* Value
Stroke/SEE	<1.0	625	1.99	Reference	–	2,057	1.50	Reference	–
	≥1.0	103	2.87	1.10 (0.35-3.46)	0.864	232	1.99	1.23 (0.58-2.65)	0.588
Major bleeding	<1.0	625	0.69	Reference	–	2,057	1.08	Reference	–
	≥1.0	103	2.25	2.48 (0.43-14.21)	0.309	232	1.48	1.23 (0.51-2.97)	0.652
ICH	<1.0	625	0.34	Reference	–	2,057	0.77	Reference	–
	≥1.0	103	0.56	–a	–a	232	1.23	1.62 (0.60-4.43)	0.343
CV events	<1.0	625	8.85	Reference	–	2,057	5.29	Reference	–
	≥1.0	103	16.36	1.30 (0.80-2.10)	0.292	232	12.64	1.91 (1.37-2.66)	<0.001
CV death	<1.0	625	1.45	Reference	–	2,057	0.66	Reference	–
	≥1.0	103	3.33	1.00 (0.29-3.38)	0.995	232	3.65	3.54 (1.75-7.18)	<0.001
All-cause death	<1.0	625	3.75	Reference	–	2,057	2.19	Reference	–
	≥1.0	103	13.32	2.41 (1.36-4.25)	0.003	232	9.75	3.20 (2.14-4.78)	<0.001
	≥1.0	103	17.35	2.18 (1.33-3.57)	0.002	232	11.46	2.26 (1.60-3.19)	<0.001

Sex, age, body mass index, history of major bleeding, type of AF, hypertension, severe liver function disorder, diabetes mellitus, hyperuricemia, heart failure/left ventricular systolic dysfunction, myocardial infarction, cerebrovascular disease, thromboembolic disease, active cancer, dementia, falls within 1 year, catheter ablation, antiarrhythmic agents, antiplatelet agents, proton pump inhibitors, P-glycoprotein inhibitors, dyslipidemia, creatinine clearance, digestive disease, and polypharmacy were included in the model.

aHR = adjusted hazard ratio; CV = cardiovascular; ICH = intracranial hemorrhage; py = person-years; SEE = systemic embolic events; other abbreviations as in Table 1.

a Not calculated due to the low number of events.

**Table 4:** Relationships Between TAT and Clinical Outcomes Based on OAC Use

	TAT, ng/mL	Warfarin (n = 728)				DOAC (n = 2,289)			
		n	Incidence Rate, /100 py	aHR (95% CI)	*P* Value	n	Incidence Rate, /100 py	aHR (95% CI)	*P* Value
Stroke/SEE	≤3	682	1.92	Reference	–	2,142	1.52	Reference	–
	>3	46	5.08	2.17 (0.61-7.64)	0.230	147	1.92	1.38 (0.54-3.50)	0.500
Major bleeding	≤3	682	0.79	Reference	–	2,142	1.14	Reference	–
	>3	46	2.43	14.03 (1.50-131.06)	0.021	147	0.76	0.63 (0.15-2.68)	0.532
ICH	≤3	682	0.32	Reference	–	2,142	0.82	Reference	–
	>3	46	1.21	–a	–a	147	0.76	1.12 (0.26-4.92)	0.878
CV events	≤3	682	9.55	Reference	–	2,142	5.73	Reference	–
	>3	46	13.92	1.44 (0.72-2.89)	0.302	147	9.49	1.60 (1.04-2.46)	0.033
CV death	≤3	682	1.65	Reference	–	2,142	0.76	Reference	–
	>3	46	2.39	0.34 (0.04-2.74)	0.309	147	3.78	4.49 (2.04-9.92)	<0.001
All-cause death	≤3	682	4.73	Reference	–	2,142	2.56	Reference	–
	>3	46	9.58	1.45 (0.63-3.32)	0.386	147	8.31	2.85 (1.74-4.64)	<0.001

Confounding variables included in the model are as listed in Table 3.

Abbreviations as in Tables 1, 2, and 3.

a Not calculated due to the low number of events.

**Table 5:** Relationships Between F1+2 and Clinical Outcomes Based on OAC Use

	F1+2 (pmol/L)	Warfarin (n = 728)				DOAC (n = 2,289)			
		n	Incidence Rate, /100 py	HR (95% CI)	*P* Value	n	Incidence Rate, /100 py	HR (95% CI)	*P* Value
Stroke/SEE	<69	165	2.22	1.96 (0.71-5.44)	0.195	8	6.83	6.66 (0.84-53.09)	0.073
	69 to <229	480	1.60	Reference	-	1,585	1.34	Reference	-
	≥229	83	4.92	2.58 (0.87-7.70)	0.089	696	1.95	1.54 (0.91-2.59)	0.105
Major bleeding	<69	165	0.00	0.00 (-, -)	-	8	0.00	0.00 (-, -)	-
	69 to <229	480	0.91	Reference	-	1,585	0.97	Reference	-
	≥229	83	2.74	3.53 (0.64-19.44)	0.148	696	1.48	1.47 (0.80-2.71)	0.212
ICH	<69	165	0.00	0.00 (-, -)	-	8	0.00	0.00 (-, -)	-
	69 to <229	480	0.34	Reference	-	1,585	0.67	Reference	-
	≥229	83	1.36	(-, -)	-	696	1.16	1.84 (0.90-3.76)	0.095
CV events	<69	165	5.96	0.87 (0.51-1.49)	0.611	8	6.83	1.27 (0.17-9.66)	0.818
	69 to <229	480	9.93	Reference	-	1,585	5.28	Reference	-
	≥229	83	18.06	1.23 (0.74-2.07)	0.425	696	7.55	1.31 (1.00-1.71)	0.0496
CV death	<69	165	0.00	0.00 (-, -)	-	8	0.00	0.00 (-, -)	-
	69 to <229	480	1.92	Reference	-	1,585	0.70	Reference	-
	≥229	83	4.02	0.92 (0.27-3.13)	0.888	696	1.54	1.79 (0.93-3.45)	0.080
All-cause death	<69	165	0.94	0.21 (0.06-0.70)	0.011	8	0.00	0.00 (-, -)	-
	69 to <229	480	5.66	Reference	-	1,585	2.39	Reference	-
	≥229	83	10.04	1.18 (0.61-2.29)	0.614	696	4.15	1.47 (1.01-2.13)	0.044

Confounding variables included in the model are as listed in Table 3.

Abbreviations as in Tables 1, 2, and 3.

**Table 6:** Relationships Between SFMC and Clinical Outcomes Based on OAC Use

	SFMC	Warfarin (n = 728)				DOAC (n = 2,289)			
		n	Incidence Rate, /100 py	aHR (95% CI)	*P* Value	n	Incidence Rate, /100 py	aHR (95% CI)	*P* Value
Stroke/SEE	−	613	1.85	Reference	–	1,823	1.52	Reference	–
	+	112	3.63	1.60 (0.62-4.15)	0.333	463	1.62	1.05 (0.57-1.93)	0.879
Major bleeding	−	613	0.61	Reference	–	1,823	1.05	Reference	–
	+	112	2.58	6.37 (1.31-30.99)	0.022	463	1.39	1.27 (0.64-2.50)	0.489
ICH	−	613	0.17	Reference	–	1,823	0.76	Reference	–
	+	112	1.54	–a	–a	463	1.04	1.38 (0.62-3.04)	0.428
CV events	−	613	9.03	Reference	–	1,823	5.71	Reference	–
	+	112	14.78	1.69 (1.06-2.72)	0.029	463	7.02	1.04 (0.77-1.41)	0.782
CV death	−	613	1.39	Reference	–	1,823	0.90	Reference	–
	+	112	3.53	2.08 (0.74-5.85)	0.167	463	1.15	1.04 (0.49-2.19)	0.925
All-cause death	−	613	4.35	Reference	–	1,823	2.69	Reference	–
	+	112	8.58	1.64 (0.89-3.01)	0.110	463	3.78	1.22 (0.81-1.83)	0.346

Confounding variables included in the model are as listed in Table 3.

Abbreviations as in Tables 1, 2, and 3.

a Not calculated due to the low number of events.

**Figure 1:**
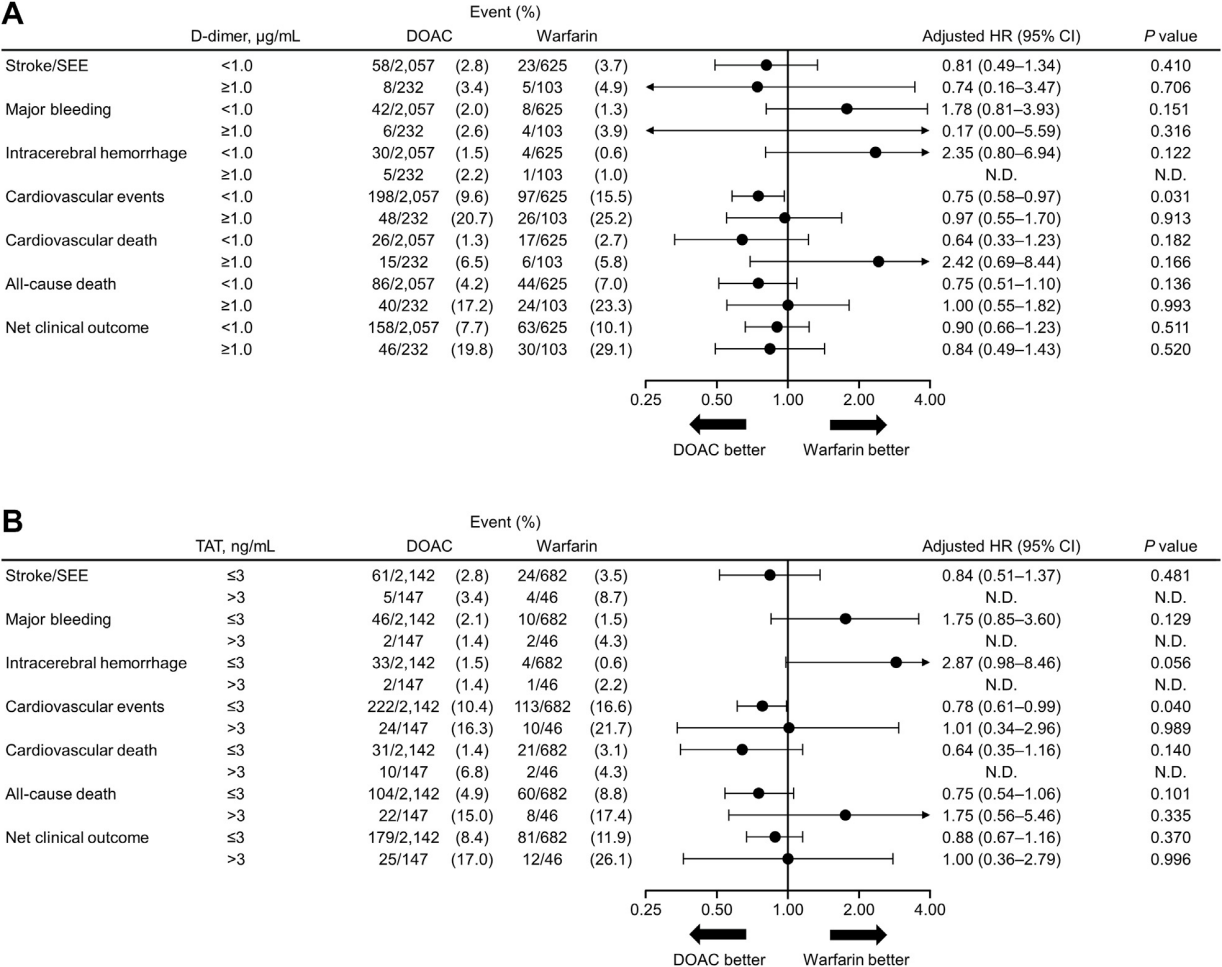

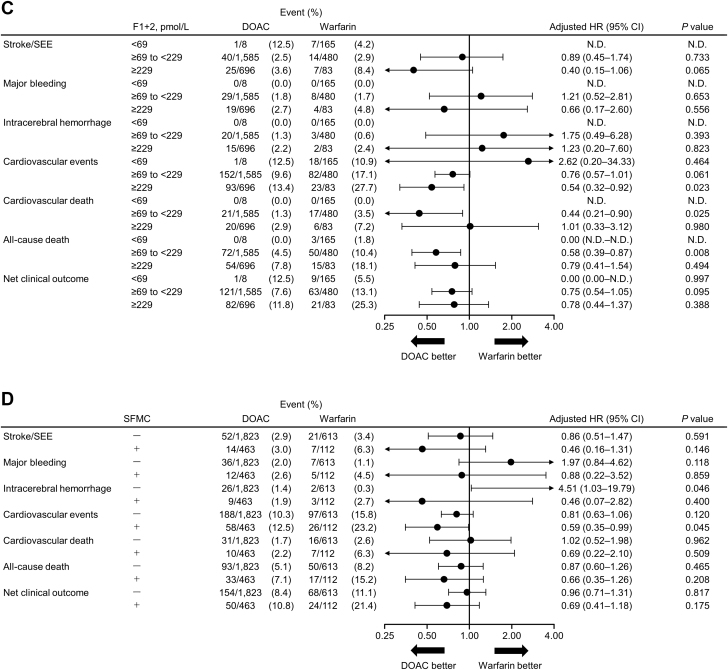
Comparison Between DOAC and Warfarin for Outcomes by Biomarkers The forest plots show **(A)** D-dimer, **(B)** TAT, **(C)** F1+2, and **(D)** SFMC. DOAC = direct oral anticoagulant; F1+2 = prothrombin fragment 1+2; SFMC = soluble fibrin monomer complex; SEE = systemic embolic events; TAT = thrombin–antithrombin complex.

Figure 1 compares DOAC vs warfarin groups for outcomes by biomarkers. As compared with patients taking warfarin, risk of CV events was lower in patients taking DOAC and with D-dimer <1.0 μg/mL (aHR: 0.75; 95% CI: 0.58-0.97; *P* = 0.031), TAT ≤3 ng/mL (aHR: 0.78; 95% CI: 0.61-0.99; *P* = 0.040), and who were SFMC positive (aHR: 0.59; 95% CI: 0.35-0.99; *P* = 0.045). A significantly higher risk for ICH was noted in SFMC-negative patients on DOACs vs warfarin (aHR: 4.51; 95% CI: 1.03-19.79; *P* = 0.046).

Supplemental Tables 1 to 4 show the factors that are associated with high levels of each biomarker, with significant associations in a multivariate analysis including male, age ≥85 years, body mass index ≥25.0 kg/m^2^, hyperuricemia, heart failure/left ventricular systolic dysfunction, no catheter ablation, and creatinine clearance <50 mL/min for D-dimer; heart failure/left ventricular systolic dysfunction and digestive disease for SFMC; polypharmacy for TAT; and female sex, age ≥85 years, no antiarrhythmic agents, no dyslipidemia, and creatinine clearance <50 mL/min for F1+2. A significant association was also found between low levels of TAT and F1+2 and long-standing persistent or permanent AF, whereas no significant difference and no consistent trend was found for D-dimer and SFMC.

## Discussion

This subcohort study aimed to clarify the relationship between coagulation biomarkers and clinical outcomes in elderly patients with NVAF. Biomarker levels of D-dimer, TAT, F1+2, and SFMC at enrollment were lower in the DOAC and warfarin groups than in the No-OAC group, and broadly similar between the DOAC and warfarin groups. Higher biomarker levels were generally associated with worse clinical outcomes, although some differences were noted between patients receiving DOACs and warfarin.

AF has been associated with elevated levels of D-dimer,^17^, F1+2,^18^ and TAT,^19^^,^^20^ suggesting activation of the coagulation pathway. In the DOAC group, higher levels of D-dimer (≥1.0 μg/mL) and TAT (>3 ng/mL) were significantly associated with increased incidences of CV events, CV death, and all-cause death, and higher levels of F1+2 (≥229 pmol/L) were significantly associated with increased incidences of CV events and all-cause death, suggesting that high activity of the coagulation pathway may contribute to these clinical events. In the warfarin group, higher levels of D-dimer were significantly associated with an increased incidence of all-cause death, higher levels of TAT with an increased incidence of major bleeding, lower levels of F1+2 with a lower risk of all-cause death, and a positive SFMC result with increased incidences of major bleeding and CV events. Among SFMC-positive patients, there was a tendency for many patients to have a prothrombin time − international normalized ratio of ≥2.6, which could explain the higher incidence of major bleeding. The mean of time in therapeutic range (TTR) was 75.9% ± 30.2% in SFMC-negative patients and 68.7% ± 31.5% in SFMC-positive patients (*P* = 0.033), whereas that in patients with TAT ≤3 ng/mL was 75.1% ± 30.1% and TAT >3 ng/mL was 69.3% ± 36.2% (*P* = 0.248). The relative risk of clinical outcomes for DOAC vs warfarin was broadly consistent regardless of biomarker levels. We observed a significantly increased risk of ICH in patients treated with DOACs vs warfarin in patients reported to be SFMC negative at enrollment. This is in contrast to results from previous large phase 3 studies of DOACs^21^^,^^22^ and the main analysis of the ANAFIE Registry (n = 32,275).^15^ It is unclear why this result was observed in the subgroup of SFMC-negative patients; however, it should be noted that the incidence of ICH of warfarin-treated patients in this subcohort study (5/725; 0.69%) was lower than that in the main study of the ANAFIE Registry (156/8,233; 1.89%) and the number of ICH events in this subgroup was small. Therefore, further analysis in a larger study would be required to confirm this result.

In the present subcohort study, a significant association between higher D-dimer levels and all-cause death was observed. These findings are consistent with those observed in the ARISTOTLE and ENGAGE AF-TIMI 48 studies, which also reported that higher D-dimer levels were associated with an increased risk of death.^9^^,^^12^ In ARISTOTLE, the benefits of apixaban compared with warfarin were consistent, regardless of the D-dimer level.^9^ The relationship between D-dimer level and the incidence of stroke/SEE in this study is not consistent with studies. In the warfarin group in the present study, the outcome might be greatly influenced by the international normalized ratio control achieved during the study; this may explain why biomarker levels at the initial stage did not seem to correlate. The findings suggest that the increase in major bleeding that was observed with increasing biomarker levels may have been related to TTR. In both ARISTOTLE and ENGAGE AF-TIMI 48, higher D-dimer levels were associated with an increased incidence of stroke/SEE. Similar results were also observed in 2 studies from Japan in patients with NVAF.^13^^,^^14^ However, in the present study, no association between D-dimer level and thromboembolic events was observed.

In the DOAC group of the present study, a higher D-dimer level was associated with a 2-fold increased risk for CV events and a 3.5-fold increased risk for CV death compared with lower levels of D-dimer. This is in agreement with the ARISTOTLE trial results, where higher D-dimer levels were associated with a higher rate of cardiac death.^9^ In those taking DOACs, although biomarkers in the trough were associated with CV events and mortality, they were not necessarily associated with stroke/SEE. Temporary increases in biomarker levels in troughs may be indicators of CV events rather than indicators of stroke events. In terms of the other coagulation biomarkers, this subcohort study identified a relationship between TAT, F1+2, and SFMC and clinical outcomes in elderly patients with NVAF for the first time.

Warfarin-treated elderly patients with NVAF are known to have an increased risk of stroke and major bleeding, despite good control.^23^^,^^24^ In other words, balancing efficacy and safety is especially important when prescribing anticoagulant therapy in patients with NVAF aged 75 years or older. Even for DOAC treatment, the balance among dose, safety, and efficacy is more important in elderly patients, who may have decreased renal function and lower body weight compared with younger patients.^22^^,^^25^ In contrast with previous studies, this subcohort study analyzed patients receiving DOACs and warfarin separately because the modes of action of these agents are different. Of note, this study indicated that the relationships between coagulation biomarkers and clinical outcomes were different between the DOAC and warfarin groups. Therefore, we suggest that when coagulation biomarkers are included in risk assessments for patients with NVAF, they should be evaluated separately for patients taking DOACs or warfarin.

In this study, heart failure was associated with high levels of D-dimer and a positive SFMC result. One possible explanation for this is that in patients with heart failure, stasis and endothelial dysfunction could trigger the coagulation pathway that consequently leads to fibrin formation.^26^ Renal dysfunction was also associated with higher levels of D-dimer and F1+2. Because D-dimer is excreted by the kidneys, decreased renal function may contribute to increased serum levels of D-dimer.^27^^,^^28^ Renal dysfunction is also typically known to be associated with a state of chronic hypercoagulation. An association between lower levels of TAT and F1+2 and the presence of long-standing persistent or permanent AF was observed. However, it is unclear why this would be the case given that long-standing persistent/permanent AF was an independent risk factor for stroke/SEE and all-cause death in the main analysis of the ANAFIE registry.^15^ It is possible that patients with paroxysmal AF have lower adherence to medication, which, in turn, might account for the differences by AF type observed in this study. However, this is only a possibility because adherence data were not recorded in the present subcohort study.

Integrating biomarkers into clinical decision-making in AF could enable precision medicine to be applied to the prescription of anticoagulants. Efforts to add biomarkers to traditional risk-score assessments have previously been reported, and this may improve the accuracy of risk assessment in patients with NVAF. For example, D-dimer has been shown to have an additional predictive value for clinical risk scores in patients with AF.^12^ Furthermore, cardiac biomarkers, such as N-terminal pro–B-type natriuretic peptide and high-sensitivity cardiac troponin used in a biomarker-based stroke risk score, consistently performed better in risk prediction and stratification than the CHA_2_DS_2_-VASc score alone, thereby offering improved decision support in patients with AF.^29^ More accurate risk assessment could ensure that anticoagulant therapy is prescribed for patients who would benefit, while avoiding unnecessary risk exposure in other patients.^12^ There is a need for further research to confirm the results of the present study for the assessment of event risk in elderly patients with NVAF.

### Study Limitations

The ANAFIE Registry had some limitations. The findings cannot be generalized to the general population because the registry targeted a specific group of Japanese elderly patients who were able to visit the study centers. Furthermore, the analysis did not consider OAC changes during follow-up, and TTR for warfarin was determined during the 6 months before enrollment. Additionally, the study design allowed participation of not only patients with newly diagnosed AF or new anticoagulant users, but also those with established NVAF or those receiving anticoagulants before enrollment. Similar to that noted in many observational studies, the proportions of patients lost to follow-up and who withdrew consent were relatively high. Other limitations were the lower-than-expected incidence of major bleeding and that the multivariate analysis was performed with only those factors that were expected to be associated with each event based on the current guidelines. Biomarker values were only examined at enrollment and not before or after the onset of an event. Pretreatment biomarker data were not collected because the ANAFIE Registry was an observational study in which many patients who were already receiving OACs were enrolled. In total, 4.9% of patients in the study were not using any OAC; however, the biomarker data in patients who were not prescribed OACs may be important because these data reflect the real-world management of elderly patients with NVAF. Differences in some patient characteristics between the groups should also be considered when interpreting the results. The higher levels of D-dimer and F1+2 at enrollment in the DOAC group vs the warfarin group may be at least partially explained by the fact that patients at a higher risk would likely have higher levels of coagulation biomarkers at enrollment. However, there may also be differences related to the mechanism of action of DOACs and the timing of blood collection (trough period); for example, the anticoagulant activity of DOACs is lowest during the trough period,^30^ so it is conceivable that the level of coagulation biomarkers was increased for that reason. Therefore, the interpretation of these results is limited because of the timing of blood collection.

## Conclusions

Blood levels of coagulation biomarkers were lower in patients treated with OACs compared with patients not receiving OACs. Elevated biomarker levels were associated with an increased risk of poor clinical outcomes, and the relationships differed between patients taking DOACs and those taking warfarin. The relative risk of clinical outcomes in patients taking DOACs vs warfarin was consistent, regardless of biomarker level. The findings suggest that using these biomarkers may improve the evaluation of prognosis for outcomes other than stroke/SEE. Future studies comparing patients on treatment with elevated vs reduced coagulation biomarkers to determine predictors of elevated residual thrombotic risk and outcomes will further our understanding of how biomarkers can improve outcome prediction.

Perspectives **COMPETENCY IN MEDICAL KNOWLEDGE:** The results of the present subcohort study will aid clinicians in choosing an appropriate treatment to achieve optimal anticoagulation with minimal complications in elderly patients with NVAF. Higher coagulation biomarker levels could help in identifying those at risk for clinical outcomes, except stroke/systemic embolic events. Considering biomarkers during clinical decision-making in AF may play a crucial role in precision medicine. The addition of certain biomarkers to traditional risk scoring systems could enhance the prediction of event risks in NVAF patients. Explicit risk assessment may allow for anticoagulant therapy for patients who would benefit, while avoiding unnecessary risk exposure for others, especially elderly patients.

**TRANSLATIONAL OUTLOOK:** Future studies should focus on including patients of all age groups and must account for OAC changes during longer follow-up periods to assess the efficacy and safety of long-term anticoagulant therapy in patients with AF.

## Funding Support and Author Disclosures

This research was supported by Daiichi Sankyo Co, Ltd. Dr Koretsune has received remuneration from Daiichi Sankyo, Bristol Myers Squibb, and Nippon Boehringer Ingelheim. Dr Yamashita has received research funding from Bristol Myers Squibb, Bayer, and Daiichi Sankyo; manuscript fees from Daiichi Sankyo and Bristol Myers Squibb; and remuneration from Daiichi Sankyo, Bayer, Pfizer Japan, and Bristol Myers Squibb. Dr Akao has received research funding from Bayer and Daiichi Sankyo; and remuneration from Bristol Myers Squibb, Nippon Boehringer Ingelheim, Bayer, and Daiichi Sankyo. Dr Atarashi has received remuneration from Daiichi Sankyo. Dr Ikeda has received research funding from Daiichi Sankyo and Bayer; and remuneration from Daiichi Sankyo, Bayer, and Pfizer Japan. Dr Okumura has received remuneration from Nippon Boehringer Ingelheim, Daiichi Sankyo, Johnson & Johnson, and Medtronic. Dr Shimizu has received research funding from Bristol Myers Squibb, Daiichi Sankyo, and Nippon Boehringer Ingelheim; and remuneration from Daiichi Sankyo, Pfizer Japan, Bristol Myers Squibb, Bayer, and Nippon Boehringer Ingelheim. Dr Suzuki has received research funding from Mitsubishi-Tanabe and Daiichi Sankyo; and remuneration from Bristol Myers Squibb and Daiichi Sankyo. Dr Tsutsui has received research funding from Daiichi Sankyo and Nippon Boehringer Ingelheim; remuneration from Daiichi Sankyo, Bayer, Nippon Boehringer Ingelheim, and Pfizer Japan; scholarship funding from Daiichi Sankyo; and consultancy fees from Pfizer Japan, Bayer, and Nippon Boehringer Ingelheim. Dr Toyoda has received lecture honoraria from Daiichi-Sankyo, Otsuka, Novartis, Abbott, Bayer, and Bristol Myers Squibb outside the submitted work. Dr Hirayama has participated in a course endowed by Boston Scientific Japan; has received research funding from Daiichi Sankyo and Bayer; and remuneration from Bayer, Daiichi Sankyo, Bristol Myers Squibb, and Nippon Boehringer Ingelheim. Dr Yasaka has received research funding from Nippon Boehringer Ingelheim; and remuneration from Nippon Boehringer Ingelheim, Daiichi Sankyo, Bayer, Bristol Myers Squibb, and Pfizer Japan. Dr Yamaguchi has acted as an Advisory Board member of Daiichi Sanky;o and received remuneration from Daiichi Sankyo and Bristol Myers Squibb. Dr Teramuki has received research funding from Nippon Boehringer Ingelheim; and remuneration from Daiichi Sankyo, Sanofi, Takeda, Chugai Pharmaceutical, Solasia Pharma, Bayer, Sysmex, Nipro, NapaJen Pharma, Gunze, Kaneka, Kringle Pharma, and Atworking. Drs Kimura, Morishima, and Takita are employees of Daiichi Sankyo. Dr Inoue has received remuneration from Daiichi Sankyo, and Bristol Myers Squibb; and consultancy fees from Daiichi Sankyo. All other authors have reported that they have no relationships relevant to the contents of this paper to disclose.
