# Supplementary material for: Coagulation Biomarkers and Clinical Outcomes in Elderly Patients With Nonvalvular Atrial Fibrillation: ANAFIE Subcohort Study
Source: JACC Asia. 2023 Aug 15;3(4):595–607. doi: 10.1016/j.jacasi.2023.06.004 (PMC10442884; doi:10.1016/j.jacasi.2023.06.004)
Supplement: Supplemental Tables 1–4 [file mmc1.docx]

**Supplemental Table 1.** Factors that influence D-dimer levels

| **Factors** | | **D-dimer, μg/mL** | | | |
| --- | --- | --- | --- | --- | --- |
|  |  | **≥1.0**  **(n = 386)** | **<1.0**  **(n = 2788)** | **Multivariate** | |
|  |  |  |  | **OR (95% CI）** | ***P* value** |
| Sex | Male* | 236 (12.7) | 1626 (87.3) | - |  |
|  | Female | 150 (11.4) | 1162 (88.6) | 0.70 (0.55, 0.90) | 0.005 |
| Age, years | <85* | 223 (9.4) | 2139 (90.6) | - |  |
|  | **≥**85 | 163 (20.1) | 649 (79.9) | 1.82 (1.41, 2.34) | <0.001 |
| Body mass index, kg/m^2^ | <18.5 | 30 (15.9) | 159 (84.1) | 1.14 (0.72, 1.80) | 0.565 |
|  | 18.5 to <25.0* | 210 (11.2) | 1659 (88.8) | - |  |
|  | **≥**25.0 | 109 (13.0) | 730 (87.0) | 1.37 (1.05, 1.80) | 0.022 |
| History of major bleeding | Yes | 20 (14.0) | 123 (86.0) | 0.93 (0.55, 1.58) | 0.792 |
|  | No* | 366 (12.1) | 2665 (87.9) | - |  |
| AF type | Paroxysmal* | 153 (11.9) | 1133 (88.1) | - |  |
|  | Persistent | 59 (12.6) | 409 (87.4) | 1.03 (0.73, 1.45) | 0.886 |
|  | Long-standing persistent/Permanent | 174 (12.3) | 1246 (87.7) | 0.82 (0.63, 1.06) | 0.135 |
| Systolic blood pressure, mmHg | <130* | 217 (12.9) | 1459 (87.1) | - |  |
|  | 130 to <140 | 69 (10.7) | 578 (89.3) | 0.77 (0.57, 1.04) | 0.089 |
|  | **≥**140 | 82 (11.7) | 616 (88.3) | 0.98 (0.73, 1.30) | 0.865 |
| Severe liver disorder | Yes | 6 (25.0) | 18 (75.0) | 2.78 (1.00, 7.74) | 0.050 |
|  | No* | 380 (12.1) | 2770 (87.9) | - |  |
| Diabetes mellitus | Yes (HbA1c <6.0%) | 17 (11.6) | 130 (88.4) | 0.85 (0.49, 1.48) | 0.572 |
|  | Yes (HbA1c **≥**6.0%) | 93 (14.1) | 567 (85.9) | 1.21 (0.91, 1.61) | 0.185 |
|  | No* | 258 (11.5) | 1982 (88.5) | - |  |
| Hyperuricemia | Yes | 134 (17.9) | 615 (82.1) | 1.49 (1.15, 1.93) | 0.003 |
|  | No* | 252 (10.4) | 2173 (89.6) | - |  |
| Heart failure/left ventricular systolic dysfunction | Yes | 199 (16.0) | 1043 (84.0) | 1.34 (1.04, 1.71) | 0.021 |
|  | No* | 187 (9.7) | 1745 (90.3) | - |  |
| Myocardial infarction | Yes | 30 (16.8) | 149 (83.2) | 1.06 (0.67, 1.68) | 0.813 |
|  | No* | 356 (11.9) | 2639 (88.1) | - |  |
| Cerebrovascular disease | Yes | 104 (13.2) | 682 (86.8) | 1.11 (0.85, 1.45) | 0.425 |
|  | No* | 282 (11.8) | 2106 (88.2) | - |  |
| Thrombosis/embolism-related disease | Yes | 47 (13.9) | 291 (86.1) | 0.92 (0.64, 1.33) | 0.650 |
|  | No* | 339 (12.0) | 2497 (88.0) | - |  |
| Active cancer | Yes | 39 (11.5) | 300 (88.5) | 0.91 (0.63, 1.32) | 0.624 |
|  | No* | 347 (12.2) | 2488 (87.8) | - |  |
| Dementia | Yes | 49 (18.4) | 218 (81.6) | 1.17 (0.81, 1.68) | 0.401 |
|  | No* | 337 (11.6) | 2570 (88.4) | - |  |
| Fall within 1 year | Yes | 44 (15.7) | 236 (84.3) | 1.29 (0.89, 1.86) | 0.175 |
|  | No* | 280 (11.3) | 2188 (88.7) | - |  |
| Anticoagulants | Warfarin* | 103 (14.1) | 625 (85.9) | - |  |
|  | No-OAC | 51 (32.5) | 106 (67.5) | 4.28 (2.74, 6.69) | <0.001 |
|  | DOAC | 232 (10.1) | 2057 (89.9) | 0.90 (0.69, 1.18) | 0.453 |
| Catheter ablation | Yes | 11 (5.3) | 196 (94.7) | 0.43 (0.23, 0.82) | 0.010 |
|  | No* | 375 (12.6) | 2592 (87.4) | - |  |
| Antiarrhythmic agents | Yes | 210 (11.6) | 1604 (88.4) | 0.91 (0.72, 1.14) | 0.402 |
|  | No* | 176 (12.9) | 1184 (87.1) | - |  |
| Antiplatelet agent | Yes | 100 (16.2) | 518 (83.8) | 1.25 (0.93, 1.68) | 0.134 |
|  | No* | 286 (11.2) | 2270 (88.8) | - |  |
| Proton pump inhibitors | Yes | 143 (12.6) | 996 (87.4) | 1.00 (0.78, 1.28) | 0.996 |
|  | No* | 243 (11.9) | 1792 (88.1) | - |  |
| P-glycoprotein inhibitors | Yes | 7 (9.2) | 69 (90.8) | 0.67 (0.29, 1.54) | 0.344 |
|  | No* | 379 (12.2) | 2719 (87.8) | - |  |
| Dyslipidemia | Yes | 166 (12.0) | 1212 (88.0) | 0.84 (0.66, 1.08) | 0.168 |
|  | No* | 220 (12.2) | 1576 (87.8) | - |  |
| Creatinine clearance, mL/min | <30, Severe renal dysfunction, dialysis | 103 (25.6) | 299 (74.4) | 2.57 (1.74, 3.79) | <0.001 |
|  | 30 to <50 | 138 (12.6) | 955 (87.4) | 1.46 (1.07, 1.99) | 0.017 |
|  | **≥**50* | 87 (7.8) | 1026 (92.2) | - |  |
| Digestive disease | Yes | 106 (11.3) | 830 (88.7) | 0.77 (0.59, 1.00) | 0.053 |
|  | No* | 280 (12.5) | 1958 (87.5) | - |  |
| Polypharmacy | <5* | 75 (8.7) | 786 (91.3) | - |  |
|  | **≥**5 | 308 (13.5) | 1969 (86.5) | 1.28 (0.94, 1.75) | 0.117 |

Data are n (%).

*Reference

All the factors shown in the column were included as an adjustment factor in the multivariate analysis model.

*Abbreviations:* AF, atrial fibrillation; CI, confidence interval; DOAC, direct oral anticoagulant; OR, odds ratio.

**Supplemental Table 2.** Factors that influence TAT levels

| **Factors** | | **TAT, ng/mL** | | | |
| --- | --- | --- | --- | --- | --- |
|  |  | **>3**  **(n = 217)** | **≤3**  **(n = 2957)** | **Multivariate** | |
|  |  |  |  | **OR (95% CI）** | ***P* value** |
| Sex | Male* | 128 (6.9) | 1734 (93.1) | - |  |
|  | Female | 89 (6.8) | 1223 (93.2) | 0.89 (0.65, 1.21) | 0.454 |
| Age, years | <85* | 145 (6.1) | 2217 (93.9) | - |  |
|  | **≥**85 | 72 (8.9) | 740 (91.1) | 1.33 (0.95, 1.86) | 0.092 |
| Body mass index, kg/m^2^ | <18.5 | 14 (7.4) | 175 (92.6) | 1.00 (0.55, 1.82) | 0.988 |
|  | 18.5 to <25.0* | 128 (6.8) | 1741 (93.2) | - |  |
|  | **≥**25.0 | 53 (6.3) | 786 (93.7) | 1.05 (0.74, 1.49) | 0.780 |
| History of major bleeding | Yes | 11 (7.7) | 132 (92.3) | 1.08 (0.56, 2.10) | 0.814 |
|  | No* | 206 (6.8) | 2825 (93.2) | - |  |
| AF type | Paroxysmal* | 100 (7.8) | 1186 (92.2) | - |  |
|  | Persistent | 41 (8.8) | 427 (91.2) | 1.18 (0.79, 1.76) | 0.414 |
|  | Long-standing persistent/Permanent | 76 (5.4) | 1344 (94.6) | 0.67 (0.48, 0.94) | 0.021 |
| Systolic blood pressure, mmHg | <130* | 134 (8.0) | 1542 (92.0) | - |  |
|  | 130 to <140 | 38 (5.9) | 609 (94.1) | 0.68 (0.47, 1.01) | 0.058 |
|  | **≥**140 | 37 (5.3) | 661 (94.7) | 0.64 (0.44, 0.94) | 0.023 |
| Severe liver disorder | Yes | 3 (12.5) | 21 (87.5) | 1.73 (0.48, 6.33) | 0.405 |
|  | No* | 214 (6.8) | 2936 (93.2) | - |  |
| Diabetes mellitus | Yes (HbA1c <6.0%) | 7 (4.8) | 140 (95.2) | 0.66 (0.30, 1.46) | 0.301 |
|  | Yes (HbA1c **≥**6.0%) | 43 (6.5) | 617 (93.5) | 0.93 (0.64, 1.35) | 0.699 |
|  | No* | 163 (7.3) | 2077 (92.7) | - |  |
| Hyperuricemia | Yes | 63 (8.4) | 686 (91.6) | 1.35 (0.96, 1.90) | 0.081 |
|  | No* | 154 (6.4) | 2271 (93.6) | - |  |
| Heart failure/left ventricular systolic dysfunction | Yes | 89 (7.2) | 1153 (92.8) | 0.97 (0.71, 1.33) | 0.860 |
|  | No* | 128 (6.6) | 1804 (93.4) | - |  |
| Myocardial infarction | Yes | 12 (6.7) | 167 (93.3) | 0.81 (0.42, 1.56) | 0.536 |
|  | No* | 205 (6.8) | 2790 (93.2) | - |  |
| Cerebrovascular disease | Yes | 49 (6.2) | 737 (93.8) | 0.86 (0.61, 1.23) | 0.406 |
|  | No* | 168 (7.0) | 2220 (93.0) | - |  |
| Thrombosis/embolism-related disease | Yes | 19 (5.6) | 319 (94.4) | 0.77 (0.46, 1.30) | 0.329 |
|  | No* | 198 (7.0) | 2638 (93.0) | - |  |
| Active cancer | Yes | 19 (5.6) | 320 (94.4) | 0.77 (0.47, 1.27) | 0.310 |
|  | No* | 198 (7.0) | 2637 (93.0) | - |  |
| Dementia | Yes | 24 (9.0) | 243 (91.0) | 1.19 (0.74, 1.92) | 0.464 |
|  | No* | 193 (6.6) | 2714 (93.4) | - |  |
| Fall within 1 year | Yes | 20 (7.1) | 260 (92.9) | 1.01 (0.61, 1.66) | 0.982 |
|  | No* | 162 (6.6) | 2306 (93.4) | - |  |
| Anticoagulants | Warfarin* | 46 (6.3) | 682 (93.7) | - |  |
|  | No-OAC | 24 (15.3) | 133 (84.7) | 2.94 (1.67, 5.19) | <0.001 |
|  | DOAC | 147 (6.4) | 2142 (93.6) | 1.11 (0.78, 1.60) | 0.555 |
| Catheter ablation | Yes | 17 (8.2) | 190 (91.8) | 1.12 (0.65, 1.94) | 0.683 |
|  | No* | 200 (6.7) | 2767 (93.3) | - |  |
| Antiarrhythmic agents | Yes | 123 (6.8) | 1691 (93.2) | 1.00 (0.75, 1.34) | 0.976 |
|  | No* | 94 (6.9) | 1266 (93.1) | - |  |
| Antiplatelet agents | Yes | 56 (9.1) | 562 (90.9) | 1.42 (0.98, 2.04) | 0.061 |
|  | No* | 161 (6.3) | 2395 (93.7) | - |  |
| Proton pump inhibitors | Yes | 87 (7.6) | 1052 (92.4) | 1.12 (0.82, 1.52) | 0.471 |
|  | No* | 130 (6.4) | 1905 (93.6) | - |  |
| P-glycoprotein inhibitors | Yes | 7 (9.2) | 69 (90.8) | 1.30 (0.58, 2.94) | 0.525 |
|  | No* | 210 (6.8) | 2888 (93.2) | - |  |
| Dyslipidemia | Yes | 86 (6.2) | 1292 (93.8) | 0.75 (0.56, 1.02) | 0.071 |
|  | No* | 131 (7.3) | 1665 (92.7) | - |  |
| Creatinine clearance, mL/min | <30, Severe renal dysfunction, dialysis | 39 (9.7) | 363 (90.3) | 1.38 (0.83, 2.30) | 0.218 |
|  | 30 to <50 | 75 (6.9) | 1018 (93.1) | 1.11 (0.76, 1.62) | 0.577 |
|  | **≥**50* | 62 (5.6) | 1051 (94.4) | - |  |
| Digestive disease | Yes | 62 (6.6) | 874 (93.4) | 0.89 (0.64, 1.23) | 0.471 |
|  | No* | 155 (6.9) | 2083 (93.1) | - |  |
| Polypharmacy | <5* | 38 (4.4) | 823 (95.6) | - |  |
|  | **≥**5 | 172 (7.6) | 2105 (92.4) | 1.78 (1.19, 2.67) | 0.005 |

Data are n (%).

*Reference

All the factors shown in the column were included as an adjustment factor in the multivariate analysis model.

*Abbreviations:* AF, atrial fibrillation; CI, confidence interval; DOAC, direct oral anticoagulant; OR, odds ratio; TAT, thrombin–antithrombin complex

**Supplemental Table 3.** Factors that influence F1+2 levels

| **Factors** | | **F1+2, pmol/L** | | | | |
| --- | --- | --- | --- | --- | --- | --- |
|  |  | **≥229**  **(n = 898)** | **<229**  **(n = 2276)** | **Multivariate** | | |
|  |  |  |  | **OR (95% CI）** | ***P* value** |  |
| Sex | Male* | 459 (24.7) | 1403 (75.3) | - |  |  |
|  | Female | 439 (33.5) | 873 (66.5) | 1.28 (1.07, 1.53) | 0.008 |  |
| Age, years | <85* | 587 (24.9) | 1775 (75.1) | - |  |  |
|  | **≥**85 | 311 (38.3) | 501 (61.7) | 1.59 (1.30, 1.95) | <0.001 |  |
| Body mass index, kg/m^2^ | <18.5 | 55 (29.1) | 134 (70.9) | 0.81 (0.56, 1.17) | 0.260 |  |
|  | 18.5 to <25.0* | 539 (28.8) | 1330 (71.2) | - |  |  |
|  | **≥**25.0 | 220 (26.2) | 619 (73.8) | 0.99 (0.80, 1.21) | 0.891 |  |
| History of major bleeding | Yes | 42 (29.4) | 101 (70.6) | 0.95 (0.63, 1.45) | 0.828 |  |
|  | No* | 856 (28.2) | 2175 (71.8) | - |  |  |
| AF type | Paroxysmal * | 450 (35.0) | 836 (65.0) | - |  |  |
|  | Persistent | 126 (26.9) | 342 (73.1) | 0.69 (0.53, 0.89) | 0.005 |  |
|  | Long-standing persistent/Permanent | 322 (22.7) | 1098 (77.3) | 0.59 (0.49, 0.72) | <0.001 |  |
| Systolic blood pressure, mmHg | <130* | 455 (27.1) | 1221 (72.9) | - |  |  |
|  | 130 to <140 | 178 (27.5) | 469 (72.5) | 0.94 (0.76, 1.18) | 0.617 |  |
|  | **≥**140 | 217 (31.1) | 481 (68.9) | 1.13 (0.91, 1.40) | 0.263 |  |
| Severe liver disorder | Yes | 7 (29.2) | 17 (70.8) | 0.81 (0.30, 2.19) | 0.672 |  |
|  | No* | 891 (28.3) | 2259 (71.7) | - |  |  |
| Diabetes mellitus | Yes (HbA1c <6.0%) | 54 (36.7) | 93 (63.3) | 1.51 (1.02, 2.23) | 0.038 |  |
|  | Yes (HbA1c **≥**6.0%) | 178 (27.0) | 482 (73.0) | 1.10 (0.88, 1.37) | 0.416 |  |
|  | No* | 622 (27.8) | 1618 (72.2 | - |  |  |
| Hyperuricemia | Yes | 214 (28.6) | 535 (71.4) | 1.14 (0.92, 1.41) | 0.226 |  |
|  | No* | 684 (28.2) | 1741 (71.8) | - |  |  |
| Heart failure/left ventricular systolic dysfunction | Yes | 352 (28.3) | 890 (71.7) | 0.93 (0.77, 1.12) | 0.445 |  |
|  | No* | 546 (28.3) | 1386 (71.7) | - |  |  |
| Myocardial infarction | Yes | 43 (24.0) | 136 (76.0) | 0.80 (0.54, 1.20) | 0.282 |  |
|  | No* | 855 (28.5) | 2140 (71.5) | - |  |  |
| Cerebrovascular disease | Yes | 209 (26.6) | 577 (73.4) | 0.88 (0.71, 1.08) | 0.207 |  |
|  | No* | 689 (28.9) | 1699 (71.1) | - |  |  |
| Thrombosis/embolism-related disease | Yes | 103 (30.5) | 235 (69.5) | 1.21 (0.91, 1.61) | 0.191 |  |
|  | No* | 795 (28.0) | 2041 (72.0) | - |  |  |
| Active cancer | Yes | 96 (28.3) | 243 (71.7) | 0.94 (0.72, 1.24) | 0.677 |  |
|  | No* | 802 (28.3) | 2033 (71.7) | - |  |  |
| Dementia | Yes | 83 (31.1) | 184 (68.9) | 0.87 (0.64, 1.19) | 0.385 |  |
|  | No* | 815 (28.0) | 2092 (72.0) | - |  |  |
| Fall within 1 year | Yes | 89 (31.8) | 191 (68.2) | 1.19 (0.89, 1.60) | 0.235 |  |
|  | No* | 671 (27.2) | 1797 (72.8) | - |  |  |
| Anticoagulants | Warfarin* | 83 (11.4) | 645 (88.6) | - |  |  |
|  | No-OAC | 119 (75.8) | 38 (24.2) | 27.34 (17.26, 43.29) | <0.001 |  |
|  | DOAC | 696 (30.4) | 1593 (69.6) | 3.99 (3.07, 5.19) | <0.001 |  |
| Catheter ablation | Yes | 64 (30.9) | 143 (69.1) | 0.96 (0.68, 1.34) | 0.794 |  |
|  | No* | 834 (28.1) | 2133 (71.9) | - |  |  |
| Antiarrhythmic agents | Yes | 463 (25.5) | 1351 (74.5) | 0.68 (0.57, 0.81) | <0.001 |  |
|  | No* | 435 (32.0) | 925 (68.0) | - |  |  |
| Antiplatelet agents | Yes | 177 (28.6) | 441 (71.4) | 0.99 (0.78, 1.26) | 0.964 |  |
|  | No* | 721 (28.2) | 1835 (71.8) | - |  |  |
| Proton pump inhibitors | Yes | 334 (29.3) | 805 (70.7) | 1.06 (0.89, 1.28) | 0.508 |  |
|  | No* | 564 (27.7) | 1471 (72.3) | - |  |  |
| P-glycoprotein inhibitors | Yes | 21 (27.6) | 55 (72.4) | 0.72 (0.41, 1.27) | 0.2591 |  |
|  | No* | 877 (28.3) | 2221 (71.7) | - |  |  |
| Dyslipidemia | Yes | 382 (27.7) | 996 (72.3) | 0.79 (0.66, 0.95) | 0.012 |  |
|  | No* | 516 (28.7) | 1280 (71.3) | - |  |  |
| Creatinine clearance, mL/min | <30, Severe renal dysfunction, dialysis | 153 (38.1) | 249 (62.0) | 2.28 (1.65, 3.14) | <0.001 |  |
|  | 30 to <50 | 335 (30.6) | 758 (69.4) | 1.46 (1.18, 1.82) | <0.001 |  |
|  | **≥**50* | 252 (22.6) | 861 (77.4) | - |  |  |
| Digestive disease | Yes | 294 (31.4) | 642 (68.6) | 1.10 (0.91, 1.34) | 0.303 |  |
|  | No* | 604 (27.0) | 1634 (73.0) | - |  |  |
| Polypharmacy | <5* | 232 (26.9) | 629 (73.1) | - |  |  |
|  | **≥**5 | 656 (28.8) | 1621 (71.2) | 1.24 (1.00, 1.54) | 0.051 |  |

Data are n (%).

*Reference

All the factors shown in the column were included as an adjustment factor in the multivariate analysis model.

*Abbreviations:* AF, atrial fibrillation; CI, confidence interval; DOAC, direct oral anticoagulant; F1+2, prothrombin fragment 1+2; OR, odds ratio.

**Supplemental Table 4.** Factors that influence SFMC levels

| **Factors** | | **SFMC, ng/mL** | | | | |
| --- | --- | --- | --- | --- | --- | --- |
|  |  | **+**  **(n = 610)** | **-**  **(n = 2558)** | **Multivariate** | | |
|  |  |  |  | **OR (95% CI）** | ***P* value** |  |
| Sex | Male* | 350 (18.8) | 1508 (81.2) | - |  |  |
|  | Female | 260 (19.8) | 1050 (80.2) | 0.99 (0.81, 1.20) | 0.906 |  |
| Age, years | <85* | 435 (18.4) | 1924 (81. 6) | - |  |  |
|  | **≥**85 | 175 (21.6) | 634 (78.4) | 1.04 (0.83, 1.29) | 0.757 |  |
| Body mass index, kg/m^2^ | <18.5 | 38 (20.1) | 151 (79.9) | 0.96 (0.65, 1.41) | 0.835 |  |
|  | 18.5 to <25.0* | 357 (19.2) | 1507 (80.8) | - |  |  |
|  | **≥**25.0 | 153 (18.2) | 686 (81.8) | 0.97 (0.78, 1.21) | 0.793 |  |
| History of major bleeding | Yes | 28 (19.6) | 115 (80.4) | 0.97 (0.62, 1.50) | 0.881 |  |
|  | No* | 582 (19.2) | 2443 (80.8) | - |  |  |
| AF type | Paroxysmal * | 250 (19.5) | 1034 (80.5) | - |  |  |
|  | Persistent | 87 (18.6) | 381 (81.4) | 0.90 (0.68, 1.19) | 0.464 |  |
|  | Long-standing persistent/ Permanent | 273 (19.3) | 1143 (80.7) | 0.88 (0.72, 1.08) | 0.234 |  |
| Systolic blood pressure, mmHg | <130* | 334 (20.0) | 1338 (80.0) | - |  |  |
|  | 130 to <140 | 94 (14.6) | 551 (85.4) | 0.70 (0.54, 0.90) | 0.006 |  |
|  | **≥**140 | 147 (21.1) | 551 (78.9) | 1.13 (0.91, 1.42) | 0.277 |  |
| Severe liver disorder | Yes | 3 (12.5) | 21 (87.5) | 0.77 (0.22, 2.63) | 0.672 |  |
|  | No* | 607 (19.3) | 2537 (80.7) | - |  |  |
| Diabetes mellitus | Yes (HbA1c <6.0%) | 35 (23.8) | 112 (76.2) | 1.20 (0.80, 1.81) | 0.381 |  |
|  | Yes (HbA1c **≥**6.0%) | 138 (20.9) | 522 (79.1) | 1.13 (0.90, 1.43) | 0.299 |  |
|  | No* | 417 (18.6) | 1819 (81.4) | - |  |  |
| Hyperuricemia | Yes | 163 (21.8) | 585 (78.2) | 1.12 (0.90, 1.40) | 0.317 |  |
|  | No* | 447 (18.5) | 1973 (81.5) | - |  |  |
| Heart failure/left ventricular systolic dysfunction | Yes | 285 (23.0) | 955 (77.0) | 1.43 (1.17, 1.75) | <0.001 |  |
|  | No* | 325 (16.9) | 1603 (83.1) | - |  |  |
| Myocardial infarction | Yes | 35 (19.6) | 144 (80.4) | 0.92 (0.61, 1.39) | 0.702 |  |
|  | No* | 575 (19.2) | 2414 (80.8) | - |  |  |
| Cerebrovascular disease | Yes | 155 (19.8) | 628 (80.2) | 1.01 (0.81, 1.25) | 0.945 |  |
|  | No* | 455 (19.1) | 1930 (80.9) | - |  |  |
| Thrombosis/embolism-related disease | Yes | 69 (20.4) | 269 (79.6) | 0.97 (0.72, 1.31) | 0.843 |  |
|  | No* | 541 (19.1) | 2289 (80.9) | - |  |  |
| Active cancer | Yes | 62 (18.3) | 277 (81.7) | 0.88 (0.66, 1.19) | 0.418 |  |
|  | No* | 548 (19.4) | 2281 (80.6) | - |  |  |
| Dementia | Yes | 63 (23.6) | 204 (76.4) | 1.19 (0.87, 1.63) | 0.278 |  |
|  | No* | 547 (18.9) | 2354 (81.1) | - |  |  |
| Fall within 1 year | Yes | 51 (18.3) | 227 (81.7) | 0.85 (0.61, 1.18) | 0.328 |  |
|  | No* | 493 (20.0) | 1971 (80.0) | - |  |  |
| Anticoagulants | Warfarin* | 112 (15.4) | 613 (84.6) | - |  |  |
|  | No-OAC | 35 (22.3) | 122 (77.7) | 1.66 (1.06, 2.61) | 0.026 |  |
|  | DOAC | 463 (20.3) | 1823 (79.7) | 1.44 (1.14, 1.82) | 0.002 |  |
| Catheter ablation | Yes | 32 (15.5) | 175 (84.5) | 0.76 (0.51, 1.13) | 0.176 |  |
|  | No* | 578 (19.5) | 2383 (80.5) | - |  |  |
| Antiarrhythmic agents | Yes | 342 (18.9) | 1469 (81.1) | 0.88 (0.73, 1.06) | 0.191 |  |
|  | No* | 268 (19.7) | 1089 (80.3) | - |  |  |
| Antiplatelet agents | Yes | 120 (19.4) | 498 (80.6) | 0.95 (0.74, 1.23) | 0.714 |  |
|  | No* | 490 (19.2) | 2060 (80.8) | - |  |  |
| Proton pump inhibitors | Yes | 210 (18.5) | 927 (81.5) | 0.84 (0.69, 1.03) | 0.087 |  |
|  | No* | 400 (19.7) | 1631 (80.3) | - |  |  |
| P-glycoprotein inhibitors | Yes | 13 (17.3) | 62 (82.7) | 0.81 (0.44, 1.50) | 0.505 |  |
|  | No* | 597 (19.3) | 2496 (80.7) | - |  |  |
| Dyslipidemia | Yes | 271 (19.7) | 1104 (80.3) | 1.00 (0.82, 1.22) | 0.982 |  |
|  | No* | 339 (18.9) | 1454 (81.1) | - |  |  |
| Creatinine clearance, mL/min | <30, Severe renal dysfunction, dialysis | 91 (22.7) | 310 (77.3) | 1.29 (0.92, 1.80) | 0.141 |  |
|  | 30 to <50 | 234 (21.4) | 859 (78.6) | 1.22 (0.97, 1.54) | 0.092 |  |
|  | **≥**50* | 190 (17.1) | 922 (82.9) | - |  |  |
| Digestive disease | Yes | 203 (21.7) | 731 (78.3) | 1.24 (1.01, 1.51) | 0.040 |  |
|  | No* | 407 (18.2) | 1827 (81.8) | - |  |  |
| Polypharmacy | <5* | 143 (16.7) | 714 (83.3) | - |  |  |
|  | **≥**5 | 459 (20.2) | 1816 (79.8) | 1.16 (0.92, 1.47) | 0.213 |  |

Data are n (%).

*Reference

All the factors shown in the column were included as an adjustment factor in the multivariate analysis model.

*Abbreviations:* AF, atrial fibrillation; CI, confidence interval; DOAC, direct oral anticoagulant; OR, odds ratio; SFMC, soluble fibrin monomer complex.
